# Skeletal muscle protein metabolism in the elderly: Interventions to counteract the 'anabolic resistance' of ageing

**DOI:** 10.1186/1743-7075-8-68

**Published:** 2011-10-05

**Authors:** Leigh Breen, Stuart M Phillips

**Affiliations:** 1 Department of Kinesiology, McMaster University, Hamilton, Ontario, Canada

## Abstract

Age-related muscle wasting (sarcopenia) is accompanied by a loss of strength which can compromise the functional abilities of the elderly. Muscle proteins are in a dynamic equilibrium between their respective rates of synthesis and breakdown. It has been suggested that age-related sarcopenia is due to: i) elevated basal-fasted rates of muscle protein breakdown, ii) a reduction in basal muscle protein synthesis (MPS), or iii) a combination of the two factors. However, basal rates of muscle protein synthesis and breakdown are unchanged with advancing healthy age. Instead, it appears that the muscles of the elderly are resistant to normally robust anabolic stimuli such as amino acids and resistance exercise. Ageing muscle is less sensitive to lower doses of amino acids than the young and may require higher quantities of protein to acutely stimulate equivalent muscle protein synthesis above rest and accrue muscle proteins. With regard to dietary protein recommendations, emerging evidence suggests that the elderly may need to distribute protein intake evenly throughout the day, so as to promote an optimal *per *meal stimulation of MPS. The branched-chain amino acid leucine is thought to play a central role in mediating mRNA translation for MPS, and the elderly should ensure sufficient leucine is provided with dietary protein intake. With regards to physical activity, lower, than previously realized, intensity high-volume resistance exercise can stimulate a robust muscle protein synthetic response similar to traditional high-intensity low volume training, which may be beneficial for older adults. Resistance exercise combined with amino acid ingestion elicits the greatest anabolic response and may assist elderly in producing a 'youthful' muscle protein synthetic response provided sufficient protein is ingested following exercise.

## Introduction

Demographics indicate that the world's population aged 60 years and over will more than triple within 50 years from 600 million in the year 2000, to more than 2 billion by 2050 [1]. As a result of this the fastest growing sub-population of society, in the developed world, is adults aged ≥ 80 [1]. This presents issues for health care for which we may well be ill-prepared. For example, aging is accompanied by numerous increasingly prevalent clinical conditions such as rheumatoid- and osteo-arthritis, vascular disease, Type II diabetes, and osteoporosis that require extensive health care resources, which can lessen the quality of life, and reduce independence [2].

Contributing to the risk of these diseases or as a direct predictor of disability itself, is the slow and inevitable age-related decline in skeletal muscle mass, referred to as sarcopenia [2]. Age related sarcopenia, particularly of type II muscle fibres, is accompanied by a decline in strength which has consequences for physical mobility/function and is associated with a greater incidence of falls in the elderly [2-4]. Although we will all lose muscle mass as we age, individual differences in: i) the rate of loss in muscle mass, ii) the age at which muscle mass starts to decline, and iii) an individual's peak muscle mass, determine the impact sarcopenia has on functional ability. Age-related sarcopenia begins in our 4-5^th ^decade and proceeds at ~0.6% annually thereafter [5]. Such a rate of sarcopenic muscle mass loss would not likely have overly dire consequences; however, during periods of muscle disuse/unloading that occur with increasing frequency in the elderly, for example, due to illness or hospitalization, the rate of sarcopenic muscle loss is exacerbated [6]. Following such periods of disuse, even something as benign as a reduction in daily step counts [6], can accelerate sarcopenic muscle loss, from which it is more difficult for the elderly to recover [7]. Thus, there is a clear need to improve our understanding of the nature of age-related sarcopenia, in order to develop non-pharmacological therapeutic interventions to improve quality of life in the elderly.

Although the etiology of sarcopenia is multifaceted [8], in the context of this review we will focus, primarily, on the contribution of alterations in muscle protein turnover (muscle protein synthesis and breakdown) to muscle wasting in the elderly. Our understanding of the contribution of muscle protein turnover to sarcopenia has shifted from a thesis in which basal muscle protein metabolism was thought to be compromised in the elderly, to a new paradigm whereby the synthetic responsiveness of muscle protein synthesis to anabolic stimuli, such as food and contractile loading, is blunted with aging. We draw on recent evidence to outline feeding and exercise interventions that may maximize the muscle protein synthetic response in elderly, possibly even to a level typically found in younger adults, thereby counteracting age-related sarcopenia.

### Muscle protein metabolism in the elderly

Skeletal muscle proteins are constantly and simultaneously synthesized and degraded. Net protein balance is defined as the difference between skeletal muscle protein synthesis (MPS) and breakdown (MPB). Thus, a significant rise in MPS (anabolism) and/or a reduction in MPB (catabolism), such that net protein balance remains positive can result in the accretion of skeletal muscle proteins. Conversely, a negative net protein balance, arising from a reduction in MPS and/or increase in MPB, will result in a loss of skeletal muscle protein. Net protein balance is maintained by ingestion of protein-containing meals which results in systemic hyperaminoacidemia that is stimulatory for the synthesis of new proteins [9-13]. Thus, in young adults consuming adequate protein and energy who are not performing exercise, skeletal muscle protein mass remains relatively stable. However, the feeding-stimulated rise in MPS is only transient and, even in the face of available amino acids, returns to basal levels [9,10]. In addition to protein consumption, exercise increases rates of MPS, thereby improving net protein balance [14-17]. The synergistic effect of protein ingestion with exercise potentiates the muscle synthetic response, swinging net balance in favour of muscle protein accretion [18,19], thereby permitting muscle hypertrophy when practiced frequently over time [20,21].

Early studies into the role of protein turnover in age-related sarcopenia reported that muscle wasting in the elderly was due to a decline in basal rates of MPS [22-24], elevated basal rates of MPB [25], or a combination of the two processes resulting in a negative net protein balance. Evidence suggested that age-related sarcopenia was the result of slower rates of basal, post-absorptive myofibrillar MPS and a more negative net protein balance in the elderly (aged >60), as compared with young adults [22-24]. Specifically, reductions in the resting fractional synthesis rates of muscle protein, on the order of 20-30%, were reported in the elderly compared with the young. In addition, others reported that surrogate markers of myofibrillar proteolysis were elevated by as much as 50% in the elderly as compared with younger adults [25]. However, if there were a simultaneous decline in resting basal FSR and an increase myofibrillar breakdown, the rate of muscle wasting would be greater than is typically observed (i.e. 0.5-1.5% per year between 50-80 years old [26]). Furthermore, a general inconsistency of findings by others to demonstrate reduced rates of MPS or markedly elevated MPB in healthy older adults [27-29], have led to the general agreement that basal skeletal muscle net protein balance is not compromised with ageing [27]. However, it is important to acknowledge that current methods used to determine muscle protein turnover may not be sensitive enough to detect very small, but potentially important, changes in muscle protein metabolism the elderly. Alternatively, the discrepant findings between these early studies with more recent papers may have been due to a failure to distinguish between healthy and frail elderly. Indeed, we postulate that basal muscle protein metabolism may be compromised in frail elderly, potentially due to greater systemic inflammation and its associated co-morbidities [30,31], or a reduction in physical activity compared with relatively 'well preserved' elderly.

**Figure 1:**
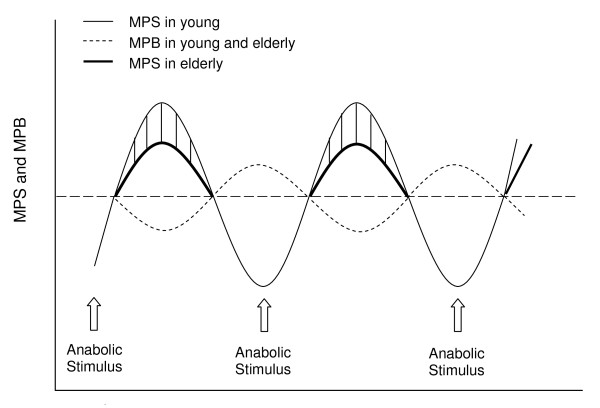
**Schematic representation of muscle protein metabolism in response to anabolic stimuli (exercise and/or amino acid ingestion) in young and elderly**. We hypothesize the primary reason for muscle loss in the healthy older adults is the inability of elderly muscles to mount a robust 'youthful' protein synthetic response to anabolic stimuli above that seen in the basal-state. Shaded section indicates the difference in MPS between elderly and young in response to anabolic stimuli.

The inconsistent findings of earlier studies, combined with evidence from older rats [32] and humans [33] showing that protein requirements may be greater with advancing age, led to the thesis that the elderly may be less able to efficiently utilize amino acids for MPS. An initial study demonstrated that basal rates of MPS were similar in the elderly and young after oral ingestion of amino acids [27]. However, the authors were unable to identify a difference in the muscle protein synthetic response to amino acids between elderly and young, despite the fact that the splanchnic extraction of amino acids was greater in the elderly (i.e. a reduction in amino acids available for delivery to skeletal muscle). However, it is possible that the muscle synthetic response was saturated as amino acids were ingested in repeated doses over several hours in this study [27]. In a follow up study, Volpi and colleagues infused subjects with an amino acid-glucose mix, bypassing splanchnic uptake, and demonstrated that rates of MPS increased in the young but not the elderly [34], which was the first demonstration of an 'anabolic resistance' of skeletal MPS in the elderly. Several years later, Cuthbertson et al. [29] were the first to compare the muscle synthetic response to bolus oral doses of crystalline essential amino acids (EAA) in the young and elderly. As hypothesized, Cuthbertson showed that basal fasted rates of MPS were indistinguishable between young and old, but MPS in the elderly was less responsive to ingestion of crystalline EAA's. The resistance of elderly muscles to a physiological dose of amino acids was subsequently confirmed by others [35-37] and is presented in Figure 1.

It is not known what causes anabolic resistance in aging muscle. Two theses are that it could be a consequence of the gradual decline in physical activity or an age-related decline in processes related to inflammation, which can interfere with protein turnover, although which of these processes plays the more prominent role in sarcopenia is difficult to answer. Given the detrimental effects of acute and chronic inflammation on skeletal muscle mass and protein metabolism, age-associated inflammation may affect the anabolic sensitivity of older muscles. To date, the strongest evidence to this effect has been generated from animal studies. For example, sepsis has been shown to induce a dramatic muscle mass loss associated with a reduction in protein synthesis rate in adult rats [38]. TNF-α, which is one of the main inducers of the acute-phase response [39], plays an important role in alterations of muscle protein metabolism in this model of sepsis [38]. Recently, others have demonstrated that low-grade inflammation (LGI) impairs the muscle protein synthetic response to feeding in rats [40]. In humans, Toth et al. [31] have shown a strong relationship exists between MPS and circulating concentrations of several markers of immune activation. At the mechanistic level, cytokines, in particularly TNF-α, may impair MPS by blunting the phosphorylation of proteins in the mammalian target of rapamycin (mTOR) intracellular signalling pathway [41]; shown to be critical for the regulation of mRNA translation and MPS for muscle hypertrophy [42].

In addition to age-related phenomenon, there is no doubt that physical inactivity *per se *has a detrimental effect on rates of MPS. In young adults, a reduction in physical activity through cast-induced immobilization of the legs blunts basal and amino-acid stimulated rates of MPS [43,44]. Thus, it is apparent that disuse induces anabolic resistance in skeletal muscle. Recent work also indicates that even short-term abrupt sedentarism, leading to a reduced relative loading of skeletal muscles, results in loss of muscle mass in the legs [7]. In addition, the cellular mechanisms underpinning the resistance of elderly muscles to amino acid provision are becoming apparent, but are far from being completely understood. Signalling proteins associated with the mTOR pathway have consistently been shown to be sensitized to amino acids [11,29]. Thus, diminished sensitivity of old muscles to anabolic stimuli may involve the impaired phosphorylation of mTOR-mediated signalling proteins. The amino acid-stimulated phosphorylation of proteins in the mTOR pathway is relatively transient, typically peaking between 1-2 h post-feeding [45] and remaining elevated above basal until ~3-4 h post-feeding [11,45]. Cuthbertson et al. [29] and Guillet et al. [46] have shown previously that the phosphorylation of mTOR and downstream targets implicated in translation initiation; ribosomal protein S6 kinase (p70S6K) and eukaryotic initiation factor 4E binding protein 1 (4E-BP1), is dampened in elderly muscles in the presence of amino acids as compared with the young. Thus, intramuscular signalling in the elderly may not be as sensitive and/or responsive to amino acids as younger muscles, and may explain the dampened MPS response older adults; further studies are required to further substantiate such a thesis, however.

The notion of anabolic resistance in elderly muscles is further supported by evidence that acute alterations in MPS (i.e. 1-3 h) after a single bout of resistance exercise [47,48] and endurance exercise [49], are impaired in the elderly as compared with young men (Figure 1). Furthermore, the hypertrophic response to a resistance exercise programme may to be impaired with ageing [50]. Drummond et al. [48] suggest that the MPS response is delayed until 3-6 h after exercise in the elderly, whereas Kumar et al. [47] have shown that MPS in the fasted state returns to basal values between 2-4 h after exercise in both the young and elderly. The discrepancies between these studies may be due, in part, to the higher volume of exercise used by Drummond et al. [48] and the fact that these subjects were studied under overnight fasted conditions. It has been demonstrated that mTOR signalling is necessary to stimulate rates of MPS after resistance exercise [51]. In combination with lower rates of MPS in the elderly after acute resistance exercise, Kumar et al. [47] showed that the phosphorylation of p70S6K and 4E-BP1 was blunted. The blunted post-exercise anabolic signalling response in the elderly was transient in nature (i.e. 1 h) and was not apparent 2 h after exercise [47]. Thus, older muscles are blunted in their capacity to mount a robust 'youthful' muscle signalling and protein synthetic response to resistance exercise. The main reason for this anabolically blunted condition with age is not fully understood and may require a greater contraction volume in older persons to achieve a more robust response.

### Protein-based interventions to counteract 'anabolic resistance'

The ingested protein dose and source dictates the amplitude and duration of the rise in EAA's in the blood, which, in turn, affects the degree of MPS [52,53]. Considerations of protein quality have obvious importance, but this may be even more important when it comes to the protein needs of older adults to maintain their muscle mass. We have shown previously in young adults that 5 g and 10 g of egg protein ingestion after resistance exercise is sufficient to acutely increase MPS above basal, a response that becomes saturated with 20 g of egg protein. Furthermore, we and others have shown, in the context of resistance exercise in young adults, that whey protein stimulates a greater acute (0-3 h post-exercise) rise in MPS compared with dose-matched casein and soy proteins [52,53], and is still highly effective at stimulating MPS over 3-5 h post-exercise [54]. The mechanism underpinning the robust anabolic properties of whey protein, relative to casein are likely related to the more rapid digestion kinetics and greater appearance of circulating amino acids [52,55], of which, the branched-chain amino acid leucine may be particularly important as a key metabolic regulator of MPS through activation of the mTOR pathway [56,57]. The superior capacity of whey to stimulate MPS over that of soy is not due to differing absorption kinetics but likely to be a mere reflection of the lower leucine content of soy versus whey protein. Thus, we are beginning to understand the required dose and amino acid source required to maximally stimulate MPS in the young.

**Figure 2:**
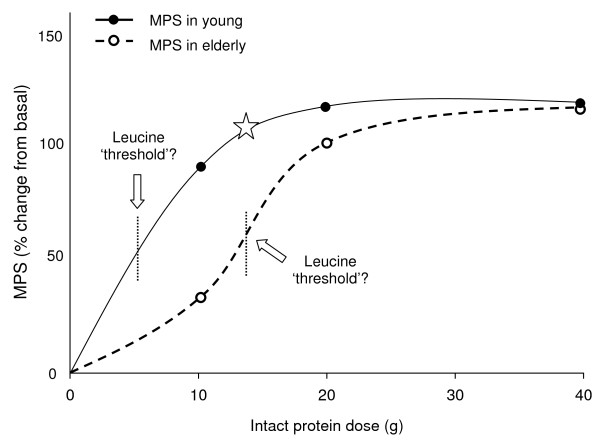
**Dose response curve of MPS in elderly and young muscle with protein ingestion at rest**. MPS in the young is stimulated above basal with ~2.5 g of crystalline EAA (found in ~5 g of intact protein) before reaching a plateau at ~10 g of crystalline EAA (found in ~20 g of intact protein). In the elderly, MPS is increased above rest after ingestion of 20 g of whey protein and, like younger adults, the response plateaus thereafter. Star indicates MPS in both young and elderly after 6.7 g of EAA (typically found in 15 g of whey protein) enriched with leucine (41% or ~2.8 g) [36]. Finely dashed lines indicate the hypothesized leucine 'threshold' which must be surpassed in order to stimulate a robust increase in rates of MPS. The threshold may be considerably lower in the young (<1 g leucine in 2.5 g of crystalline EAA's) compared with the elderly (~1.5-2 g of leucine contained in 15-20 g of whey protein).

Data concerning the protein dose required to acutely increase rates of MPS in the elderly are conflicting. Using frequent small bolus feedings, Welle and Thornton [58] suggested that low (7% total energy), moderate (14% total energy) and high protein (28% total energy) meals stimulate rates of MPS to a similar extent. Furthermore, the data of Cuthbertson et al. [29] showed that anabolic resistance to crystalline EAA ingestion was only apparent after 10-20 g doses, but as little as 2.5 g of crystalline EAA was sufficient to increase myofibrillar MPS above rest in both young and elderly. The data of Cuthbertson et al [29] suggest that in anabolically resistant elderly muscles a low dose of amino acids is sufficient to stimulate MPS above rest but that the muscle is refractory to a higher dose of EAA or protein [58]. Recent studies from our laboratory have led us to posit that the elderly may require more protein to acutely increase rates of MPS than the young. Specifically, we constructed a dose-response curve of myofibrillar MPS rates to graded doses of intact whey protein ingestion in the elderly. In line with the findings of Cuthbertson [29], we show that the basal rate of myofibrillar MPS in the elderly plateaus after the ingestion of 20 g of whey protein, containing ~10 g of EAA (Yang, Breen et al. 2011. Resistance exercise enhances myofibrillar protein synthesis with graded intakes of whey protein in older men, submitted). However, in contrast with the findings of Cuthbertson [29] who showed as little as 2.5 g of crystalline EAA increased MPS rates above rest, we show that lowest dose of 10 g of whey, containing ~5 g of EAA, did not elicit an anabolic response. Thus, we now have preliminary evidence that MPS increases in young muscles sharply after a meal containing low doses of amino acids (5-20 g), and becomes saturated after larger doses (20-40 g), whereas in the elderly the muscle protein synthetic response is blunted in response to low doses of amino acids (Figure 2).

The mechanism facilitating the impaired dose-response of MPS to amino acids in the elderly compared with the young is still elusive. However, we [59], and others [60,61] have hypothesized the existence of a leucine 'threshold' that must be surpassed after protein ingestion to stimulate MPS above rest. This threshold is not absolute and is graded with lower leucine stimulating responses in young persons, whereas the elderly become less sensitive and the threshold becomes higher. The leucine threshold hypothesis is based on a number of recent observations [36,57,60-62], the most striking of which is that leucine ingestion/infusion results in a phosphorylation of proteins critical for the mTOR pathway [57]. We postulate that young muscles are highly sensitive to the anabolic actions of leucine as ~1 g of orally ingested leucine seems to be sufficient to stimulate MPS above rest [63]. In contrast, our recent observations indicate that the elderly are less sensitive to the anabolic actions of leucine as ~2 g of leucine found in 20 g of whey protein was required to increase MPS rates above rest (Yang, Breen et al. 2011. Resistance exercise enhances myofibrillar protein synthesis with graded intakes of whey protein in older men, submitted). Thus, we suggest that the greater leucinemia associated with rapidly digested high leucine-content proteins, may be crucial in order to facilitate a robust muscle protein synthetic response in the elderly. Indeed, the recent work of Pennings et al. [53] shows the greater circulating concentrations of leucine after ingestion of whey, compared with casein protein, was associated with elevated rates of MPS. Furthermore, there was a strong relationship between peak leucinemia and MPS in this study [53]. Consistent with the thesis that leucinemia is important in 'driving' MPS, the work of Katsanos and colleagues [36] demonstrated that 6.7 g of EAA (equivalent to ~15 g of whey), increased MPS above rest in the elderly, but only after the leucine content of EAA's was increased from 26 to 41% (1.7 to 2.8 g). In line with our hypothesis that younger muscles are more anabolically sensitive, the 26% low-leucine treatment in this study was sufficient to increase MPS in the young. Thus, when considering protein feeding strategies that will acutely increase MPS in the elderly, a protein source with high leucine content and rapid digestion kinetics, in order to promote a transient leucinemia 'spike' would be an effective option.

Despite the acute anabolic effects of leucine feeding, the impact of chronic leucine supplementation on lean mass maintenance/growth in the elderly is still unclear due, in large part, to the limited number of studies. Supplementation with leucine-rich amino acid mixtures has been shown to improve strength and physical function [64,65], and increase lean mass [66,67]. A recent study by Verhoeven and colleagues [68] was the first, to our knowledge, to investigate the long-term effects of free-leucine supplementation (7.5 g·day^-1^) in the elderly. The authors showed no effect of leucine supplementation on muscle mass or strength compared with a non-supplemented placebo group. One possibility is that free-leucine simply 'turns on' mRNA translational processes, but other essential amino acids are required to facilitate the anabolic actions of leucine and promote hypertrophy. Another suggestion is that leucine supplementation may need to be provided over a much longer period (i.e. > 8 months) than has previously been studied [69]. Finally, given evidence in young adults that supplementation of leucine-rich high-quality proteins, such as whey, augment exercise-induced lean mass and strength gains to a greater extent than other protein sources [20,70], free-leucine supplementation provided in the context of a resistance exercise training programme may promote muscle hypertrophy in the elderly. Most likely, however, is that the rate of any potential leucine-enhanced gains in feeding-induced MPS to augment muscle mass are going to be too small for methods such as DXA and biopsy to detect, even over a period of 6 months [71]. At this time it is not possible to definitively conclude on the efficacy of leucine *per se *with or without concurrent exercise training to counteract or prevent sarcopenia.

In line with recent recommendations from Paddon-Jones [72], we suggest that in order to elevate rates of MPS and induce a net positive protein balance in resistant elderly muscles, it may be important for older adults to consume sufficient quantities of high-quality, leucine-rich proteins to stimulate MPS. Thus, older adults should distribute their daily protein equally across three or more daily meals. For example, given our findings that the elderly require more protein to increase MPS above rest than the young, in a 75 kg individual consuming ~60 g of protein daily (based on the RDA of 0.8 g·kg^-1^), this would mean consuming ~20 g of protein with each meal, as opposed to a typical feeding regimen in which the elderly typically ingest smaller amounts of protein with breakfast (~8 g) and lunch (~12 g) and the majority of dietary protein with dinner (~40 g) [73]. The notion of equal daily protein distribution for the elderly is based, partly, on a recent nitrogen balance study in which Campbell and colleagues [74] showed that dietary protein requirements are not different between young and old and are similar to previous daily recommendations (~0.85 g·kg^-1^·day^-1^). It is important, however, to note that slowly turning over pools of protein, such as muscle, are captured in such measurements and whether slow declines in MPS, and thus muscle size, are too small to be captured in a short-term nitrogen balance study. For example, a 30% decline in resting (from 0.03%·h^-1 ^to 0.027%·h^-1 ^for 10 of 24 h in a day) and feeding-stimulated (from 0.06%·h^-1 ^to 0.042%·h^-1 ^for 14 of 24 h in a day) myofibrillar protein synthesis would only result in a lower requirement for protein approximating 0.1 g protein·d^-1 ^(assuming a 70 kg male with 30 kg of muscle and that muscle is a homogeneous pool) which is well within the error of the nitrogen balance method.

### Resistance exercise-based interventions to counteract 'anabolic resistance'

Resistance exercise acutely increases MPS [22,29,48] and, when practiced frequently over time, promotes muscle hypertrophy in older adults [75-78], although the anabolic response is generally blunted compared with younger adults [47,79,80]. It has been suggested these divergent responses to resistance exercise may be due to the fact that the elderly generally lift a much lower volume of weights than the young [47]. However, we suggest that resistance to the anabolic nature of hypertrophic exercise may be critical in mediating the muscle adaptive remodelling in the elderly. To date, only a small number of studies have sought to manipulate contractile activity in order to optimally stimulate MPS and, in doing so, have uncovered interventions with the potential to help elderly muscles overcome anabolic resistance to exercise stimuli.

It has been shown that low-intensity resistance-exercise (20-50% 1RM), coupled with blood flow occlusion to the working muscle (using of an inflatable cuff), induces hypertrophy and strength gains similar to those found with high-intensity resistance exercise [81,82]. Specifically, Fry et al. [83] demonstrated that low-intensity leg resistance exercise (4 sets of 15-30 reps at 20% 1RM) with blood flow restriction, increased mTOR signalling phosphorylation and MPS by ~56% above rest in the elderly over 3 h post-exercise. Given that MPS in elderly muscle does not respond to traditional high-intensity resistance exercise in the same manner as young [47], low-intensity blood flow restriction resistance exercise has been touted as an alternative, novel rehabilitation therapy. Whether blood flow restriction can be implemented effectively in exercise programmes designed for the elderly remains to be seen. Taken together with the knowledge that the elderly cannot train as comfortably at the same relative high-intensity as the young, these data beg the question; how low can the resistance exercise intensity be and still produce a physiological adaptation and functional benefit? In a study by Fry et al. [83] it was shown that resistance exercise at 20% 1RM, without blood restriction, was not sufficient to increase rates of MPS above rest. A recent publication from our laboratory [84], albeit in healthy young adults, showed that low-load high volume leg resistance exercise to failure (30% of 1RM) was more effective at increasing MPS than high-load low volume resistance exercise to failure (90% of 1RM). Specifically, weight lifting at 30% of 1RM to failure induced similar increases in MPS to that found after lifting 90% of 1RM to failure at 4 h post-exercise [84]. This response was sustained at 24 h only after the low-load high volume exercise, which is in opposition to the commonly held belief that one needs to lift heavier weights to induce hypertrophy [85]. Thus, resistance exercise-induced MPS may not necessarily be load-dependant but may instead be determined by exercise volume; this would be good news for older adults who may be unable to sustain lifting weights at a relative high intensity due to sarcopenic co-morbidities, such as osteoarthritis or other connective tissue complications.

Previously, we have shown [76] that low-intensity resistance exercise training (40% of 1RM), 3 times a week for 12 weeks, increases leg strength, but not muscle thickness in the elderly, suggestive of a neuromuscular adaptation. In contrast, high-intensity resistance exercise (80% 1RM) in the elderly induced greater strength gains than the low-intensity group, accompanied by an increase in quadriceps muscle thickness [76]. Importantly, ~10 repetitions *per *set were performed by both the low and high-intensity groups in this study [76], thus the low-intensity group performed ~50% less volume (repetitions x mass). Given these data, we suggest that low-intensity resistance training be performed to fatigue, which may result in a greater load than one is able to perform with high-intensity training, in order to promote equivalent gains in lean mass and strength in the elderly [84]. This paradigm would require older adults exercising at a low-intensity to complete a higher number of repetitions, or weight lift to failure. The efficacy of long-term low-load high-intensity training programmes for inducing hypertrophy in the elderly has yet to be determined.

Finally, the synergistic anabolic effect of resistance exercise in combination with essential amino acid ingestion has been well documented in the young [19,54,86-88] and elderly [48,89-91]. In our previous publication, as little as 4.2 g of EAA (10 g of whey protein) can potentiate the exercise-induced rise in MPS in healthy young adults [87]. Thus, there can be little doubt that utilizing amino acid or protein feeding and resistance exercise concurrently will promote an optimal anabolic environment in elderly muscles compared with either stimulus alone. Drummond et al. [48] demonstrated that EAA ingestion after a bout of resistance exercise increased rates of MPS to a similar extent in young and elderly over 5 h post-exercise. In this study, an acute (1-3 h post-exercise) MPS response was only apparent in the young, but a delayed MPS response (3-5 h post-exercise) was greater in the elderly, suggesting that the combination of EAA plus resistance exercise may abolish anabolic resistance in the elderly via a delayed stimulation of MPS. Previously, we have shown that maximal post-exercise stimulation of MPS can be achieved with ingestion of 20 g of egg protein in young adults [63]. However, recent data from our laboratory (Yang, Breen et al. 2011. Resistance exercise enhances myofibrillar protein synthesis with graded intakes of whey protein in older men', submitted) suggest that post-exercise rates of MPS increase in a stepwise manner in response to graded doses of whey protein ingestion, the greatest increase apparent with 40 g of whey (40 g > 20 g > 10 g). Thus, there is discordance between this finding and our observation that, in the non-exercised basal state, 20 g of whey protein maximally stimulates rates of MPS. Thus, in the young, exercise-induced rates of MPS plateau when 20 g of high-quality protein is ingested, but in the elderly it appears that post-exercise rates of myofibrillar MPS can be increased further with ingestion of larger doses of protein. However, the feasibility of instructing older adults to ingest 40 g of protein in the post-exercise period is questionable.

Regarding the optimal timing of protein intake to maximize resistance exercise-induced muscle protein anabolism, there are few data available in the elderly [90-92]. Human data intimate that mTOR mediated signalling and MPS may be suppressed during exercise, but appear to rebound by ~60 min of recovery [91,93,94]. Thus, it has been suggested providing amino acids in the post-exercise 'window of opportunity' may induce the greatest anabolic response. However, studies have failed to demonstrate an effect of manipulating the timing of protein ingestion (i.e. pre, peri or post-exercise) on the acute synthetic response of muscle protein in the young and elderly [88,89,91,95]. Interestingly, evidence to the contrary exists in chronic training studies. For example, in older adults, Esmark et al. [92] showed that resistance training (over 12 weeks) followed by immediate ingestion of 10 g of protein resulted in greater gains in muscle cross-section and isokinetic strength, compared with a group who delayed protein intake until 2 h after each work-out. These data [92] are somewhat puzzling as the gains in lean mass with immediate post-exercise feeding were of similar magnitude to those reported by others in the elderly with no specific feeding intervention [50,77,78]. Thus, Esmarck et al. [92] did not actually demonstrate any superior anabolic responses with immediate ingestion of protein after resistance training. Furthermore, these findings are not universal as others [96] have failed to show similar long-term benefits of immediate pre- and post-exercise protein ingestion for muscle mass and strength in the elderly. Recent evidence from our laboratory albeit in healthy, young adults suggests that muscles remain sensitive to protein ingestion at 24 h after low or high-intensity resistance exercise performed to failure [97], as evidenced by an increase in mRNA translational signalling and MPS. Thus, protein feeding during late exercise recovery still maintains the capacity to be synergistic to exercise-mediated rates that have been shown to exist in the fasted-state during this time [14,98]. Whether this is the case in older adults remains to be shown. The notion that ingestion of amino acids immediately after exercise is not critical for optimal stimulation of MPS is supported by several studies [88,95,99]. Of these studies, Tipton et al. [95] provide intriguing evidence that net muscle protein synthesis is greater when an amino acid-carbohydrate mixture is ingested prior to exercise, compared with drink ingestion after exercise. Taken together, these data give equivocal insight into the most appropriate time to for older adults to consume protein to optimize the resistance exercise-induced anabolic response. It appears that protein ingestion at doses of at least 20 g and perhaps as high 30-40 g, in close proximity to, and at intervals over 24 h after resistance exercise, may be able to elicit an anabolic response in the elderly.

### Polyunsaturated fatty acids as an intervention to counteract 'anabolic resistance'

Fish-oil-derived omega-3 fatty acids (n-3 polyunsaturated fatty acids) might be a potentially useful therapeutic agent for the treatment and prevention of sarcopenia. Initial studies in animals showed that supplementation with omega-3 fatty acids increased the phosphorylation of anabolic signalling proteins, whole-body protein synthesis [100] and attenuated the loss of muscle mass in burned animals [101]. In elderly humans, Smith et al. [102] showed that 8-weeks of omega-3 fatty acid supplementation augmented hyperinsulinemic-hyperaminoacidemic induced increases in MPS and mTOR signalling, suggesting that omega-3 fatty acids attenuate age-related anabolic resistance. Whilst the exact mechanisms by which omega-3 fatty acids act on translational signalling and MPS in the elderly are not entirely clear, the anti-inflammatory properties of omega-3 fatty acids [103] may be a critical factor, particularly in the frail elderly who display elevated levels of inflammation [30]. On the other hand, recent evidence suggests that fatty acids may have intrinsic muscle protein anabolic properties, as long-chain n-3 polyunsaturated fatty acid supplementation augments hyperinsulinemic-hyperaminoacidemic stimulated muscle protein synthesis in healthy young subjects [104], in whom inflammation would be expected to be relatively low. Taken together, these data suggest that fatty acid supplementation alone is insufficient to promote an anabolic response (as basal MPS is not increased). Instead, additional stimuli, such as amino acids, may be required to permit the anabolic effect of fatty acids.

### Non-steroidal anti-inflammatory drugs to restore 'anabolic sensitivity'

The anabolic sensitivity of aged rat skeletal muscle can be restored when LGI is controlled with the non-steroidal anti-inflammatory drug (NSAID), ibuprofen [105]. In older humans, training-induced gains in muscle mass and strength appear to be greater in subjects supplemented with over-the-counter NSAID's [106] compared with non-supplemented elderly. The mechanism of NSAID action appears to involve decreasing throughput in the nuclear factor kappa B (NF*κ*B) pathway via actions consequent to decreasing cyclooxygenase 2 (COX2) activity [105]. Pro-inflammatory cytokines increase the synthesis of prostaglandin-E2 (PGE2) in many cells types [107] by activation of COX2, a rate-limiting enzyme in the synthesis of PGE2. A decrease of PGE2 production has been shown to preserve MPS and decrease MPB in skeletal muscle in highly inflamed rat models [108,109]. To date, the paucity of human studies makes it difficult to recommend a specific NSAID that will effectively counteract low-grade inflammation induced anabolic resistance. However, as highlighted by Trappe et al. [106], there are many elderly who take acetaminophen, ibuprofen and other non-prescription or prescription COX inhibitors on a regular basis, thus, the role of this class of drugs in skeletal muscle metabolism needs to be further defined. At this time, the risks associated with COX inhibitors [110,111] and the precise mechanisms underpinning the response of skeletal muscle to NSAID's need to be considered before the recommended use of these drugs in any population.

## Summary

Ageing is associated with a blunted muscle protein synthetic response to anabolic stimuli, i.e. feeding and resistance exercise. However, dietary and exercise interventions may prevent or slow sarcopenic muscle loss. First, we stress the importance of ingesting sufficient protein with each meal for older adults. Specifically, given the blunted sensitivity of older muscles to low doses of amino acids, a dietary plan that includes at least 20 g and as high as 30 g of high quality protein per meal will provide sufficient essential amino acids, particularly leucine, required to elicit a robust acute muscle protein synthetic response above that seen at rest, thereby promoting the accretion of muscle protein over time. Secondly, we advise the use of resistance exercise in the elderly to induce hypertrophy, improve strength and improve physical function. In older adults who are not restricted by physical disability, frequent high-intensity weight lifting will increase lean muscle mass. Alternatively, low-intensity high volume weight lifting may also promote an adaptive response in the elderly provided the working muscle is sufficiently 'stressed' (i.e. via blood flow restriction to the muscle or lifting to failure). Finally, utilizing resistance exercise and protein ingestion concurrently will promote an optimal anabolic response than either stimulus alone and should be an important consideration for clinicians and patients alike. However, in order to maximize the anabolic potential of prior resistance exercise, the elderly may require more protein (~40 g) than the young (~20 g). Further work is required in order to delineate the most appropriate feeding strategies to augment resistance exercise adaptation in the elderly.

## Competing interests

The authors declare that they have no competing interests.

## Authors' contributions

LB and SMP drafted, edited and approved the final manuscript.
